# Fresh versus frozen embryo transfer after gonadotropin-releasing hormone agonist trigger in gonadotropin-releasing hormone antagonist cycles among high responder women: A randomized, multi-center study

**Published:** 2018-01

**Authors:** Abbas Aflatoonian, Mahnaz Mansoori-Torshizi, Maryam Farid Mojtahedi, Behrouz Aflatoonian, Mohammaad Ali Khalili, Mohammad Hossein Amir-Arjmand, Mehrdad Soleimani, Nastaran Aflatoonian, Homa Oskouian, Nasim Tabibnejad, Peter Humaidan

**Affiliations:** 1 *Research and Clinical Center for Infertility, Yazd Reproductive Sciences Institute, Shahid Sadoughi University of Medical Sciences, Yazd, Iran.*; 2 *Novin Infertility Center, Mashhad, Iran.*; 3 *Department of Obstetrics and Gynecology, Endocrinology and Female Infertility Unit, Roointan Arash Women's Health Research and Educational Hospital, Tehran University of Medical Sciences, Tehran, Iran.*; 4 *Stem Cell Biology Research Center, Yazd Reproductive Sciences Institute, Shahid Sadoughi University of Medical Sciences, Yazd, Iran.*; 5 *Department of Reproductive Biology, School of Medicine, Shahid Sadoughi University of Medical Sciences, Yazd, Iran.*; 6 *Department of Advanced Medical Sciences and Technologies, School of Paramedicine, Shahid Sadoughi University of Medical Sciences, Yazd, Iran.*; 7 *Madar Hospital, Yazd, Iran.*; 8 *Armaghan Infertility & IVF Clinic, Mashhad, Iran.*; 9 *The Fertility Clinic, Skive Regional Hospital and Faculty of Health, Aarhus University, Aarhus, Denmark.*; * Nasim Tabibnejad and Peter Humaidan are equal corresponding authors.

**Keywords:** Fresh embryo transfer, Fresh, Frozen-thawed embryo transfer, GnRH antagonist, GnRHa trigger, OHSS, Reproductive Outcome

## Abstract

**Background:**

The use of embryo cryopreservation excludes the possible detrimental effects of ovarian stimulation on the endometrium, and higher reproductive outcomes following this policy have been reported. Moreover, gonadotropin-releasing hormone agonist trigger in gonadotropin-releasing hormone (GnRH) antagonist cycles as a substitute for standard human chorionic gonadotropin trigger, minimizes the risk of ovarian hyperstimulation syndrome (OHSS) in fresh as well as frozen embryo transfer cycles (FET).

**Objective:**

To compare the reproductive outcomes and risk of OHSS in fresh vs frozen embryo transfer in high responder patients, undergoing in vitro fertilization triggered with a bolus of GnRH agonist.

**Materials and Methods:**

In this randomized, multi-centre study, 121 women undergoing FET and 119 women undergoing fresh ET were investigated as regards clinical pregnancy as the primary outcome and the chemical pregnancy, live birth, OHSS development, and perinatal data as secondary outcomes.

**Results:**

There were no significant differences between FET and fresh groups regarding chemical (46.4% vs. 40.2%, p=0.352), clinical (35.8% vs. 38.3%, p=0.699), and ongoing (30.3% vs. 32.7%, p=0.700) pregnancy rates, also live birth (30.3% vs. 29.9%, p=0.953), perinatal outcomes, and OHSS development (35.6% vs. 42.9%, p=0.337). No woman developed severe OHSS and no one required admission to hospital.

**Conclusion:**

Our findings suggest that GnRHa trigger followed by fresh transfer with modified luteal phase support in terms of a small human chorionic gonadotropin bolus is a good strategy to secure good live birth rates and a low risk of clinically relevant OHSS development in in vitro fertilization patients at risk of OHSS.

## Introduction

With the development of assisted reproductive technology (ART) and the increase in the number of ovarian stimulation cycles, it is important to be able to manage the possible complication of excessive ovarian response to stimulation. Ovarian hyperstimulation syndrome (OHSS) still remains a life-threatening complication in ART with a reported incidence of hospitalization of 0.3% (1); however, the reporting of OHSS is a grey zone and the incidence is undoubtedly higher. One of the most effective approaches to prevent OHSS is using of gonadotropin-releasing hormone agonist (GnRHa) for final oocyte triggering. Use of GnRHa trigger as an alternative of the gold standard human chorionic gonadotropin (hCG) trigger either eliminates or significantly reduces the risk of OHSS development in GnRH antagonist cycle (2-8). 

The physiological mechanism behind this is a short and self-limiting luteotropic effect following GnRHa trigger which reduces the risk of severe OHSS, dissimilar to the continuous LH-like (luteinizing hormone) activity induced by an hCG trigger. On the other hand, the reduced luteal phase LH level leads to insufficient corpus luteum function as well as defective neo-vascularization around the implanting embryo due to down-regulation of growth factors like vascular endothelial growth factor A and fibroblast growth factor 2 (9, 10). 

Therefore, the early luteal phase decrease in circulating LH levels results in a luteal phase deficiency, negatively impacting endometrial receptivity and implantation if only a standard luteal phase support is used (10). By the introduction of the “modified luteal phase support” after GnRHa trigger, good reproductive outcomes have been reported in GnRHa triggered cycles using either supplementation with oestradiol and progesterone (3, 11) or low-dose hCG (8, 12-14).

At the same time, freeze all embryos using vitrification after GnRHa trigger has been explored and has proven to be a safe and advantageous alternative for OHSS prevention, concomitantly reducing the probable detrimental effects of controlled ovarian stimulation on the endometrium (15, 16). Thus, the freeze-all or segmentation strategy, including prolonged embryo culture to the blastocyst stage helps selecting the highest quality embryos for cryo-preservation and transfer in a subsequent cycle. By planning a freeze-all cycle, the possible harmful effects of ovarian stimulation on the endometrium may be prevented, and even higher reproductive outcomes as compared to fresh embryo transfer have been reported (16, 17).

As the number of trials using the freeze, all policy is still scarce, the aim of the current study was to compare the reproductive outcomes of fresh vs frozen embryo transfer in high responder IVF patients triggered with GnRHa. Secondly, we wanted to compare the risk of OHSS development in the women with OHSS risk who were randomized to either a fresh transfer or a freeze-all cycle.

## Materials and methods

**Subjects**

In this multicenter, randomized clinical trial, 280 infertile women at risk of OHSS were recruited at three fertility clinics in Iran; Yazd Research and Clinical Center for Infertility (n=40), Yazd Madar Hospital (n=100) and Mashhad Novin Fertility and Infertility Center (n=140) between January 2014 and January 2017. Patients with OHSS risk at the age between 20-40 yr and having a number of 14-25 follicles ≥12 mm on the day of trigger and a body mass index >18 and <35 kg/m^2^ were included in the study. Exclusion criteria were patients with less than 14 and more than 25 follicles ≥12 mm on the day of trigger, patients with a previous history of OHSS development, endocrine disorders and >40 yr of age.

**Treatment protocol**

Women who participated in the trial were stimulated with a fixed dose of recombinant human follicle stimulating hormone rFSH (Gonal-F) (150 to 225 IU) subcutaneously for the first 5 days. Serial trans-vaginal sonography was performed during stimulation. Once follicles reached the size of ≥14 mm, daily co-treatment with GnRH antagonist (Cetrotide) (Cetrorelix, Merck Serono Laboratories, Aubonne, Switzerland) (0.25 mg/daily), subcutaneously started until the day of triggering final oocyte maturation. On this day, participants were randomized to either frozen embryo transfer (FET) or fresh embryo transfer groups (n=140/each) using computer-generated random numbers in wrapped, unlabeled envelope each holding a unique number. The subjects, nurses, and physicians were not blinded to the assigned treatment group. Triggering was performed in all participants using 0.2 mg GnRHa (Decapeptyl®, 0.1 mg) subcutaneously when at least two follicles reached a mean diameter of 17 mm. Trans-vaginal oocyte retrieval was performed after 36 hr. 

In the fresh transfer group, two embryos of good or excellent quality were transferred 48-72 hr after oocyte retrieval, using an embryo transfer Labotect catheter (Labor-Technik-Göttingen GmbH, Gottingen, Germany) or a Cook (Sydney, Australia) catheter. In the fresh transfer group, 1500 IU hCG (Pregnyl, Organon, Netherland) was administered on the day of embryo transfer. Moreover, progesterone suppositories (Cyclogest®) (Cox Pharmaceuticals, Barnstaple, UK) 400 mg twice daily were administered vaginally, from the day of oocyte retrieval until the observation of fetal heart activity by ultrasound in the 8^th^ wk. 

In the FET group embryos were vitrified on day 2 after oocyte collection as previously described (18). The subsequent cycle was considered as a study cycle. 

For endometrial preparation prior to transfer in FET group, all women received oral estradiol valerate (Aburaihan Co., Tehran, Iran) 6 mg/ day from the second day of the menstrual cycle. Endometrial thickness was assessed by vaginal ultrasonography on the 14^th^ day of the cycle. When endometrial thickness reached ≥8 mm, all participants received Cyclogest vaginal pessaries (Cox Pharmaceuticals, Barnstaple, UK) 400 mg twice daily until menstruation or for the 8 wk after positive β-hCG in case of a clinical pregnancy. Embryo transfer was performed 3 days after the beginning of progesterone administration.

**Data collection**

Basal clinical and laboratory data were collected from the hospital records. Moreover, in each center, a telephone questionnaire including data on maternal and neonatal parameters was achieved by a trained nurse, based on patient information. It should be noted that the majority of cases were referred from distant cities; therefore, data on perinatal outcomes were collected, using a telephone questionnaire. Blood samples were analyzed for measurement of oestradiol and progesterone on the day of trigger. The identification criteria for developing OHSS were baseline ovarian reserve measures, including serum anti mullerian hormone level and antral follicle count followed by clinical examination and transvaginal sonography. 

As described above, the majority of participants in this study came from distant cities; therefore, it was not possible to follow signs and symptoms of OHSS observationally. Instead, patients were thoroughly informed about the disease appearances and complications and were then subsequently followed via telephone calls for subjective description. In cases, with moderate OHSS a physical examination was performed either by the participating hospitals or for distant patients by the local general practitioner or gynecologist for clarification of signs.

**Outcome parameters**

The primary outcome parameter was clinical pregnancy and the secondary outcome parameters were chemical pregnancy, live birth, OHSS development, and perinatal data including gestational age, birth weight, gender, multiple pregnancy status, stillbirth, ectopic pregnancy, and pregnancy loss. Reproductive outcomes were defined as follows; 

Clinical pregnancy: observation of fetal heart activity by transvaginal ultrasonography 2-3 wk after positive β-hCG. 

Chemical pregnancy: β-hCG >50 IU/L on day 14 after embryo transfer. 

Pregnancy loss: Loss of pregnancy before 20 wk of gestation. 

Stillbirth: Fetal death after 20 wk of gestation. 

Ectopic pregnancy: Detection of extra uterine pregnancy by repeated β-hCG and ultrasound. Laparoscopy was performed in rare cases. 

Preterm birth: Gestational age <37 wk at delivery. 

Small for gestational age: Birth weight less than 10^th^ centile for gestational age. 

Low birth weight (LBW): Infant weight <2500 gr at birth.

**OHSS classification**

Patients with signs of OHSS were divided into three classifications according to signs and symptoms. Mild OHSS was defined by ovarian enlargement, lower abdominal discomfort, mild nausea, vomiting, and abdominal distention. Worsening of symptoms, ascites, and ovarian enlargement up to 12 cm were considered as moderate OHSS. Finally, severe OHSS was characterized by severe pain, quick weight gain, tense ascites, hemodynamic instability, respiratory difficulty, progressive oliguria, and laboratory abnormalities (19).

**Ethical consideration**

The study was approved by Ethics Committee of Yazd Research and Clinical Center for Infertility, Shahid Sadoughi University of Medical Sciences, Yazd, Iran (IRCT2016092224512N4). All subjects signed a written informed consent for participation. The study is reported according to the CONSORT statement.

**Statistical analysis**

We calculated that at least a total of 280 cases are needed (140 in each group) to identify a 15% difference in the clinical pregnancy rate between FET and fresh embryo transfer cycles. A power of 80% and p<0.05 level of significance were considered for this study. Intention-to-treat (ITT) analysis was used in the comparison of chemical, clinical, and ongoing pregnancy rates, and live birth rate between the FET and fresh group and involved 240 subjects who were initially allocated into two groups. Per protocol, analyses were also applied for aforementioned variables between groups among cases who were not excluded from the study. 

The Statistical Package for the Social Science version 20 for Windows (SPSS Inc, Chicago. IL, USA) was used for data analysis. Differences between normally distributed continuous variables were measured by Student’s *t *test. Continuous variables without normal distribution analyzed using Mann-Whitney U test. The Chi-square test was used to compare categorical variables. Statistical significance was set at a p <0.05. Adverse or protective effects of FET on perinatal outcome vs fresh cycles are expressed as odds ratio

## Results

In total, 1280 women were initially assessed for enrollment. 1000 subjects were excluded failing to meet the inclusion criteria. A total of 280 women was randomized to FET (n=140) and fresh (n=140) groups. 21 women in the fresh and 19 women in the FET group never started the treatment. Therefore, 119 and 121 women received GnRHa triggering in the fresh and FET groups, respectively. Among subjects, who underwent oocyte retrieval, 10 women in the fresh transfer group were excluded because of the risk of severe OHSS development (>25 follicles on the day of the trigger). Also, 5 women in the FET group withdrew their consent to participate in the study and subsequently opted for fresh transfer. One woman in each group did not have suitable embryos for transfer and was excluded from the study. Finally, 108 and 115 women had an embryo transfer in the fresh and FET groups, respectively (Figure I). Baseline characteristics were similar in two groups. In contrast, serum estradiol level, numbers of retrieved oocytes, and mature oocytes were significantly higher in the FET group compared to the fresh transfer group (p<0.001) (Table I). 

**Reproductive outcomes**

Chemical, clinical, and ongoing pregnancy rates were comparable between two study groups (Table II). The live birth through both intention-to-treat (ITT) (27.3% for FET vs. 26.9% for fresh transfer) and per protocol analysis (30.3% for FET vs. 29.9% for fresh transfer) showed no statistical difference (p=0.947 and p=0.953). The influence of independent variables was analyzed using multivariate logistic regression analyses in the FET and fresh transfer groups (Table IV). 

**Perinatal outcomes**

No differences were seen between groups for the perinatal outcomes. Finally, no differences were seen as regards prematurity, birth defects, and sex between groups (Table V).

**OHSS**

There were no significant differences regarding OHSS between groups. In the FET group, 29.8% and 5.8% presented mild and moderate OHSS, respectively, vs 37% and 5.9%, respectively, in the fresh transfer group. No participant in both groups developed severe OHSS and no one required admission to the hospital (Table III).

**Figure 1:**
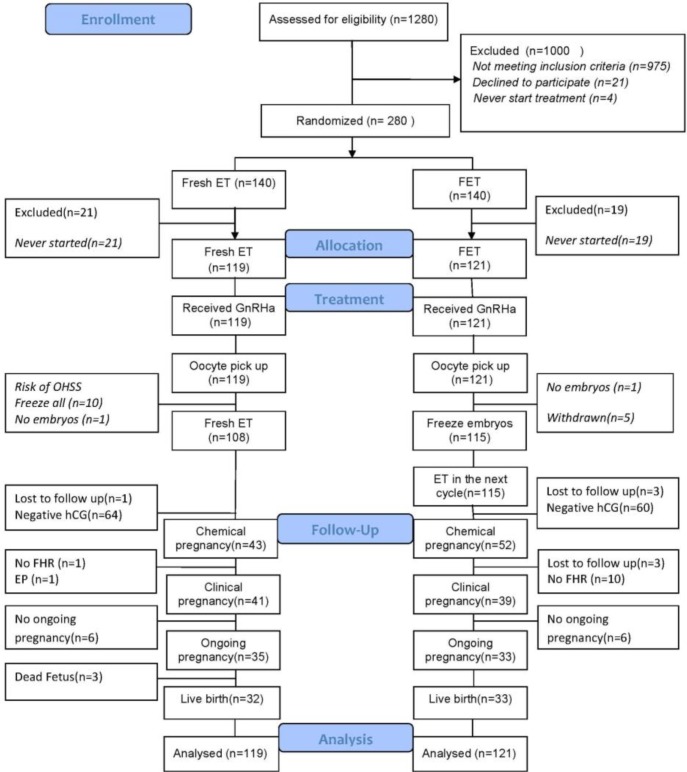
Flowchart of participants’ allocation, treatment, follow-up, and analysis

**Table I:** Baseline characteristics and cycle parameters in fresh and FET groups

**Variable**		**FET group (n= 121)**	**Fresh group (n= 119)**	**p-value**
Age (yr)*		28.84 ± 4.40	28.62 ± 4.48	0.701
BMI (kg/m^2^)*		25.54 ± 4.21	25.21 ± 3.93	0.529
Duration of infertility (yr)**		4.75 (IQR= 4)	4 (IQR= 6)	0.194
Primary cause of infertility***				
	Male factor	41 (33.9)	44 (37)	0.959
	PCOS	43 (35.5)	39 (32.8)	
	Tubal factor	17 (14)	19 (16)	
	Unexplained	7 (5.8)	6 (5)	
	Mixed	13 (10.7)	11 (9.2)	
Estradiol (pg/ml)**		3000 (IQR= 2218)	2272 (IQR= 1442)	<0.001
Progesterone on the day of trigger (ng/ml)**		1.65 (IQR= 2.11)	1.45 (IQR= 2.07)	0.741
No. of retrieved oocyte**		19 (IQR= 11)	12 (IQR= 10)	<0.001
No. of matured oocyte**		14 (IQR= 10)	10 (IQR= 8)	<0.001

Statistical analysis was carried out using Student's *t* test.

BMI: Body mass index

PCOS: Poly cystic ovary syndrome

FET: Frozen-thawed embryo transfer.

* Data are presented as mean±SD ,

** Data are presented as median (IQR: Interquartile range),

*** Data are presented as number (%)

**Table II:** Clinical outcomes in fresh vs FET group according to per protocol and intention to treat analysis

**Variable**	**Groups**		**OR (95% CI)**	**Groups**		**OR (95% CI)**	**p-value**
	**FET** **(Per protocol)**	**Fresh** **(Per protocol)**		**FET** **ITT (n= 121)**	**Fresh** **ITT (n= 119)**		
Chemical pregnancy	52/112 (46.4)	43/107 (40.2)	1.29 (0.75-2.20)	52/121 (43)	43/119 (36.1)	1.33 (0.79-2.23)	0.352* 0.279**
Clinical pregnancy	39/109 (35.8)	41/107 (38.3)	0.89 (0.51-1.55)	39/121 (32.2)	41/119 (34.5)	0.90 (0.52-1.54)	0.699* 0.715**
Ongoing pregnancy	33/109 (30.3)	35/107 (32.7)	0.89 (0.50-1.58	33/121 (27.3)	35/119 (29.4)	0.90 (0.51-1.57)	0.700* 0.713**
Live birth	33/109 (30.3)	32/107 (29.9)	1.01 (0.56-1.82)	33/121 (27.3)	32/119 (26.9)	1.02 (0.57-1.80)	0.953* 0.947**

Data presented as n/total (%).Statistical analysis was carried out using Chi-squared test.

FET: Frozen embryo transfer

OR: Odds ratio

CI: Confidence interval

ITT: Intention to treat

* p-value: Difference between FET and fresh groups in per protocol analysis

** p-value: Difference between FET and fresh groups in ITT analysis

**Table III:** OHSS occurrence in fresh vs FET group

**OHSS** **occurrence**	**FET (n=121)**	**Fresh (n=119)**	**p-value**
No OHSS	78 (64.4)	68 (57.1)	0.480
Mild	36 (29.8)	44 (37)	
Moderate	7 (5.8)	7 (5.9)	
Severe	--	--	

Data presented as n (%).

OHSS: Ovarian hyper stimulation syndrome

FET: Frozen embryo transfer

**Table IV:** Influence of independent variables on the clinical outcome using multivariate regression logistic

**Dependent variable**	**Independent variable**	**Adjusted OR (95% CI)**	**p- value**
Chemical pregnancy	Madar Hospital Yazd Infertility Center Novin Infertility Center	1(Ref) 0.14(0.06-0.32) 0.33(0.12-0.85)	0.0001
			0.0001
			0.022
	Age	0.99(0.92-1.08)	0.970
	BMI	1.10(1.01-1.19)	0.016
	Type infertility Primary Secondary	1(Ref) 1.07(0.49-2.35)	0.856
	Group FET Fresh	1(Ref) 1.10(0.57-2.14)	0.768
	Retrieved oocytes	1.06(0.99-1.14)	0.085
	Mature oocytes	0.98(0.92-1.05)	0.710
	Estradiol	0.04(1.00-1.00)	0.072
Clinical pregnancy	Madar Hospital Yazd Infertility Center Novin Infertility Center	1(Ref) 0.12(0.05-0.30) 0.30(0.11-0.80)	0.0001
			0.0001
			0.017
	Age	1.00(0.92-1.08)	0.969
	BMI	1.05(0.98-1.14)	0.145
	Type infertility Primary Secondary	1(Ref) 1.02(0.45-2.28)	0.953
	Group FET Fresh	1(Ref) 1.51(0.77-2.97)	0.228
	Retrieved oocytes	1.04(0.97-1.11)	0.205
	Mature oocytes	0.99(0.94-1.05)	0.965
	Estradiol	1.00(1.00-1.00)	0.976
Live birth	Madar Hospital Yazd Infertility Center Novin Infertility Center	1(Ref) 0.14(0.05-0.35) 0.30(0.10-0.83)	0.0001
			0.0001
			0.021
	Age	0.99(0.92-1.08)	0.979
	BMI	1.00(0.93-1.09)	0.825
	Type infertility Primary Secondary	1(Ref) 0.93(0.41-2.11)	0.875
	Group FET Fresh	1(Ref) 1.23(0.62-2.43)	0.548
	Retrieved oocytes	1.03(0.97-1.10)	0.233
	Mature oocytes	1.00(0.95-1.05)	0.895
	Estradiol	1.00(1.00-1.00)	0.620

Analysis adjusted for recruitment center, age, BMI, type of infertility, fresh embryo transfer /FET, estradiol, number of retrieved, and mature oocytes.

Statistical analysis was carried out using multivariate logistic regression.

OR: Odds Ratio

CI: Confidence interval

BMI: Body mass index

FET: Frozen-thawed embryo transfer

**Table V:** Perinatal outcomes in fresh and FET groups

**Variable**		**FET**		**Fresh**		**OR (95% CI)**		**p-value**	
Singleton pregnancy		27/33 (81.8%)		26/32 (81.2%)		1.03 (0.29-3.63)		0.953	
Twin pregnancy		6/33 (18.2%)		6/32 (18.8%)					
Prematurity		7/33 (21.2%)		6/32 (18.8%)		0.85 (0.25-2.89)		0.804	
LBW		6/33 (18.2%)		4/32 (12.5%)		1.55 (0.39-6.12)		0.526	
Anomaly at birth		5/33 (15.2%)		5/32 (15.6%)		0.96 (0.25-3.71)		0.958	
Neonatal gender									
	Girl		16/33 (48.5%)		14/32 (43.8%)		0.82 (0.31-2.19)		0.702
	Boy		17/33 (51.5%)		18/32 (56.2%)				

Statistical analysis was carried out using the Chi-squared test.

OR: Odds Ratio

CI: Confidence interval

LBW: Low birth weight

FET: Frozen-thawed embryo transfer

## Discussion

OHSS is a serious problem of assisted reproductive techniques, and optimally to reduce the risk of OHSS development the ovarian stimulation protocol should be individualized according to risk factors (20, 21). In the current multi-centric study, we compared clinical and perinatal outcomes as well as the OHSS occurrence in OHSS risk IVF/ICSI patients (15-25 follicles ≥12 mm on the day of trigger) who were randomized to either frozen embryo transfer (FET) or fresh transfer on the day of trigger GnRHa trigger. 

The study was powered for a possible difference in clinical pregnancy rate in favor of FET. However, no statistical difference was seen for clinical pregnancy rate. Moreover, chemical and ongoing pregnancies, as well as live birth rate, were similar between groups. No statistical differences were seen regarding OHSS development between groups. It was previously reported that using a low dose bolus of luteal hCG improves the clinical outcome after GnRHa trigger in OHSS risk patients (12, 20, 22, 23). Others suggested high dose oestradiol and progesterone for luteal phase support in OHSS risk patients after GnRHa trigger. This intensive luteal phase support package was also reported to result in high ongoing pregnancy rates as well as OHSS reduction (3). 

Imbar and colleagues applied a similar strategy for luteal phase support, comparing clinical outcomes after fresh and frozen-thawed embryo transfer. The authors reported similar implantation pregnancy, and live birth rate rates in both groups with no OHSS development (11). Moreover, a recent PRISMA review and meta-analysis among normo-responder patients concluded that GnRHa triggering in conjunction with modified luteal phase support, using a small bolus of hCG in addition to a standard luteal phase support, resulted in similar live birth rates compared to hCG trigger (24). This is contrasted with a previous Cochrane review claiming that despite a significant decrease in OHSS development, GnRHa trigger was associated with a lower live birth and ongoing pregnancy rate (25). 

However, and importantly, the Cochrane review included early studies in which only a standard luteal phase support without any modifications of the luteal phase support after GnRHa trigger was used, which severely flawed the conclusion of the analysis (25). In line with other previous reports in GnRHa triggered cycles (8, 20, 22), no severe OHSS was seen in our study in neither FET nor the fresh transfer group. It should be noted that 10 patients in the fresh group were excluded because of the risk of severe OHSS. Similarly, others did not report severe OHSS in individualized low dose hCG luteal support after GnRHa trigger (20, 26). Based on our results the occurrence of mild and moderate OHSS in the FET group, was 29.8% and 5.8%, respectively, while, 37% and 5.9% of patients in the fresh transfer group presented mild and moderate OHSS, respectively. Datta and coworkers reported an incidence of 16.2% for mild to moderate OHSS after GnRHa trigger compared to 31% after hCG trigger in fresh transfer cycles (22). 

In a previous randomized controlled multi centric study no OHSS was seen in the group at risk of OHSS (>14 follicles) post GnRHa trigger regardless of supplementation with a bolus of 1.500 IU hCG; in comparison an severe OHSS incidence of 3.4% was reported in the group of patients at risk of OHSS triggered with hCG (8). Regarding clinical outcomes after GnRHa trigger and FET, it was previously suggested that elective cryopreservation leads to high success rates in only one stimulated cycle (27). Vlaisavljević and colleagues vitrified all embryos in a group of high responders (≥19 follicles on trigger day) at the blastocyst stage and transferred the frozen-thawed embryos in the subsequent cycle. A total of 65.9% of patients obtained a live birth after six embryo transfer cycles. The cumulative live birth rate after six embryo transfer cycles was reported to be 76.6% with no occurrence of OHSS (27). The authors suggested that the chance of achieving a live birth was the same for women who still had cryopreserved embryos and did not return for embryo transfer compared to patients referring for embryo transfer (27). Finally, a survey compared clinical outcomes of FET and fresh transfer after hCG trigger in high responders. A significantly higher implantation, clinical and cumulative pregnancy rate in freeze-all cycles in comparison to fresh embryo transfer cycles were seen (28). Earlier we compared pregnancy and neonatal outcomes of children born after FET and fresh embryo transfer. Chemical pregnancy as well as neonatal outcomes was similar between FET and fresh embryo transfer cycles except for lower live birth rate in FET vs fresh embryo transfer group (29).

In our previous study the perinatal outcome was compared between FET and fresh embryo transfer (18). We found that prematurity was significantly increased among singleton newborns in the FET group compared to infants born after fresh embryo transfer. Moreover, the percentages of LBW infants were reduced significantly in twin and triple pregnancies in the FET compared to fresh group (18). Nevertheless, our previous findings showed no significant difference regarding LBW between FET and fresh groups as well as between singleton, twin and triple pregnancies (29). In the current study prematurity and LBW were comparable in high responder patients triggered with GnRHa in the FET and the fresh transfer groups. Furthermore, we observed a similar twinning incidence in the FET and fresh group (18.2% and 18.8%, respectively). In addition, the percentages of major and minor anomalies at birth were similar in both groups. In line with our results in current and previous (29) studies, Belva and colleagues reported the comparable rate of major congenital malformations in live born infants between the FET and the fresh group (30). 

A limitation of the present study is the absence of blinding of patients, nurses and physicians, and the fact that OHSS reporting for some patients was performed by either GP`s or gynecologists outside the study.

## Conclusion

In conclusion, in this study the clinical outcomes were similar between fresh and frozen transfer after GnRHa trigger, suggesting that GnRHa trigger followed by fresh transfer with modified luteal phase support in terms of a small hCG bolus is a good strategy to secure good live birth rates and a low risk of clinically relevant OHSS in IVF patients at risk of OHSS development.

## Note

***Registration ID in IRCT: IRCT2016092224512N4***
